# Keyword-optimized template insertion for clinical note classification via prompt-based learning

**DOI:** 10.1186/s12911-025-03071-y

**Published:** 2025-07-03

**Authors:** Eugenia Alleva, Isotta Landi, Leslee J. Shaw, Erwin Böttinger, Ipek Ensari, Thomas J. Fuchs

**Affiliations:** 1 Windreich Department of Artificial Intelligence and Human Health at Mount Sinai, Icahn School of Medicine at Mount Sinai, New York, USA; 2 Hasso Plattner Institute for Digital Health at Mount Sinai, Icahn School of Medicine at Mount Sinai, New York, USA; 3 Institute for Personalized Medicine, Icahn School of Medicine at Mount Sinai, New York, USA; 4 Blavatnik Family Women’s Health Research Institute, Icahn School of Medicine at Mount Sinai, New York, USA; 5 Wyss Center for Bio and Neuroengineering, Geneva, Switzerland

**Keywords:** NLP, Encoders, Information extraction, Dysmenorrhea, Gatortron, Prompt

## Abstract

**Background:**

Prompt-based learning involves the additions of prompts (i.e., templates) to the input of pre-trained large language models (PLMs) to adapt them to specific tasks with minimal training. This technique is particularly advantageous in clinical scenarios where the amount of annotated data is limited. This study aims to investigate the impact of template position on model performance and training efficiency in clinical note classification tasks using prompt-based learning, especially in zero- and few-shot settings.

**Methods:**

We developed a keyword-optimized template insertion method (KOTI) to enhance model performance by strategically placing prompt templates near relevant clinical information within the notes. The method involves defining task-specific keywords, identifying sentences containing these keywords, and inserting the prompt template in their vicinity. We compared KOTI with standard template insertion (STI) methods in which the template is directly appended at the end of the input text. Specifically, we compared STI with naïve tail-truncation (STI-s) and STI with keyword-optimized input truncation (STI-k). Experiments were conducted using two pre-trained encoder models, GatorTron and ClinicalBERT, and two decoder models, BioGPT and ClinicalT5, across five classification tasks, including dysmenorrhea, peripheral vascular disease, depression, osteoarthritis, and smoking status classification.

**Results:**

Our experiments revealed that the KOTI approach consistently outperformed both STI-s and STI-k in zero-shot and few-shot scenarios for encoder models, with KOTI yielding a significant 24% F1 improvement over STI-k for GatorTron and 8% for Clinical BERT. Additionally, training with balanced examples further enhanced performance, particularly under few-shot conditions. In contrast, decoder-based models exhibited inconsistent results, with KOTI showing significant improvement in F1 score over STI-k for BioGPT (+19%), but a significant drop for ClinicalT5 (−18%), suggesting that KOTI is not beneficial across all transformer model architectures.

**Conclusion:**

Our findings underscore the significance of template position in prompt-based fine-tuning of encoder models and highlights KOTI’s potential to optimize real-world clinical note classification tasks with few training examples.

**Supplementary information:**

The online version contains supplementary material available at 10.1186/s12911-025-03071-y.

## Introduction

Clinical note classification is a common clinical natural language processing (NLP) task, and often a necessary step to correctly characterize patient cohorts from electronic health records (EHRs). Recently, transformer-based language model [1], pre-trained on large clinical text corpora, have emerged as versatile NLP tools to solve multiple clinical tasks, including text classification via direct fine-tuning [2]. However, this approach is often limited by the small size of annotated datasets available in clinical scenarios. This limitation is not surprising considering that annotation requires extensive domain knowledge which, in the clinical field, is particularly expensive [3].

Prompt-based learning [2, 4–6] has recently emerged as an effective technique to adapt pretrained models with zero or only few training examples. A template is appended to a model’s input conditioning the model’s output so that it can directly be mapped to a label class through a verbalizer [6]. While several aspects of prompt design have been extensively investigated, only few studies have characterized the effect of template position. This gap is especially relevant for encoder models like BERT, which allow prompts to be placed flexibly anywhere in the text. Moreover, it is an important consideration for clinical note classification, where relevant information is often sparse or scattered due to the presence of copy-pasted text and instances included for administrative purposes [7].

In this work, we assessed the effect of template position in a zero- and few-shot prompt-based learning setting for clinical note classification. The main contributions of this work are:

- The development of a keyword-optimized template insertion method (KOTI) to identify optimal template positions and show that KOTI can improve model performance in zero- and few-shot settings.
- The superiority of KOTI over naive ‘tail’ truncation of standard template insertion approaches. Truncating the clinical note leveraging the presence of keywords improves performance, while maintaining computational efficiency.
- The improvement of model performance when training on balanced examples compared to random samples of the same size, in few-shot learning settings.

## Background

### Transformer-based language models

The Transformer model, introduced by Vaswani et al. [1], leverages the self-attention mechanism to capture long-range dependencies in the input data and facilitate parallelized training. This enabled the development of bi-directional encoder models like BERT [7], which are pre-trained using masked language modeling (MLM). In MLM, random tokens in a sentence are replaced with a special [MASK] token, and the model learns to predict these masked tokens based on the surrounding context. This process produces rich, bidirectional contextual embeddings that excel in tasks such as text classification and entity recognition. In contrast, decoder models like GPT [8] use an autoregressive training objective — they predict the next token in a sequence — which relies on unidirectional (causal) self-attention. This means that at each step, the model only attends to previously generated tokens, making it well-suited for text generation. Hybrid architectures, such as T5 [9], combine both encoder and decoder components to handle a wide array of text-to-text tasks, including translation, by leveraging the strengths of both bidirectional encoding and unidirectional attention for text generation.

### Prompt-based learning

Prompt-based learning adapts pretrained models to specific tasks with little or no additional training data [3–6], and has been successfully applied in the clinical NLP setting [8]. In encoder models (e.g., BERT), a prompt template containing a [MASK] token is appended to or embedded within the input text. The model then predicts the masked token(s), and these predictions are mapped to target labels through a verbalizer. This approach leverages the MLM objective from pretraining, allowing the model to incorporate context from both before and after the mask, which in turn produces rich, bidirectional representations.

In contrast, decoder models (e.g., GPT) typically receive a textual prompt—often in the form of a question or an incomplete sentence—and generate next tokens which can subsequently mapped to labels through a verbalizer [4]. Due to their autoregressive nature, which processes text sequentially from left to right, decoder models generally have limited flexibility for inserting prompts at arbitrary positions within the text. Instead, prompts for these models are typically positioned at the beginning or end of the input, or a combination thereof. As a result, the opportunity to dynamically adjust prompt position in decoder models is more constrained.

### Previous work

Several authors have explored the use of prompt-based learning with encoder models for clinical note classification. Sivarajkumar and Wang [9] built a framework for classifying clinical notes via prompt-based learning in a zero-shot setting, achieving high performance for ICD-10 disease codes classification tasks. Taylor et al. [10] compared several combinations of manual and soft (i.e., learnable) templates and verbalizers to classify notes using ICD-10 codes. During training, they updated prompt-related parameters while keeping the pre-trained language model frozen and reported a better performance compared to standard fine-tuning. Wang et al. [11] trained a prompt-based classifier for dementia detection. Yang et al. [12] framed a clinical note classification task using ICD-9 codes as a multilabel task, simultaneously appending one prompt template per ICD-9 code. They also included a step of knowledge injection via contrastive learning using ICD-9 hierarchical codes.

The effect of the template position on a model outcome has only been investigated by Wang et al. [11]. Authors compared appending or pre-pending the template to the input text without showing a consistent advantage of one over the other. Sivarajkumar and Wang [9] mentioned the problem of clinical note truncation due to token number limitations. In their framework, they solved this issue by splitting notes into smaller chunks, predicting labels for each chunk separately, and finally aggregating them via max pooling.

Outside of the clinical field, only few studies have investigated the effect of the template position, all of them concentrating on soft templates only. Wu et al. [13] investigated the effect of soft template position (appending vs pre-pending) for single-sentence or sentence-pair classification without splitting the original input sentence, while Mao et al. [14] also interspersed the template tokens across the sentence. Both works showed that prompt position impacts a model performance and is task dependent. Finally, Yang et al. [15] developed a dynamic prompting framework, where soft template position is optimized together with length and representation, considering only appending and prepending as insertion methods.

## Methods

### Datasets and tasks

We performed experiments on five different classifications tasks (see Table 1). We used two publicly available datasets, the N2C2 smoking challenge [16] (N = 502) dataset for smoking status classification task (Smk) and the N2C2 obesity challenge [17] dataset for peripheral vascular disease (PVD) (N = 1,115), depression (Dep) (N = 1,111), and osteoarthritis (OA) (N = 1,105) classification tasks. Both datasets consist of de-identified clinical notes (medical discharge summaries) provided by the Research Patient Data Registry at Partners Healthcare.

**Table 1:** Prompt configuration for each task

Task	Classes	Template	Label-Words	Keywords
Dys	Yes, No, Unknown	dysmenorrhea: [MASK]	yes, no, unknown	dysmenorrhea, cramps, menstrual pain, period pain
OA	Yes, Unmentioned	osteoarthritis (OA): [MASK]	yes, no	bone, osteo, arthritis, osteoarthritis, joint, cartilage, OA
Dep	Yes, Unmentioned	depression: [MASK]	yes, no	depressive, depression, mood
PVD	Yes, Unmentioned	peripheral vascular disease (PVD): [MASK]	Yes, No	vascular, peripheral vascular, arterial
Smk	current, past, no, unknown	smoking: [MASK]	yes, no, past, unknown	smoking, smoke, cigar, cigarette

Moreover, we included our own task for the classification of dysmenorrhea (Dys) from N = 300 clinical notes of gynecological encounters from the Mount Sinai Data Warehouse.

#### Dysmenorrhea classification task (Dys)

The aim of this task was to classify clinical notes for *dysmenorrhea, no dysmenorrhea,* or *unknown*. First, we randomly selected and manually annotated 300 clinical notes related to routine gynecological examinations from the Mount Sinai Health System, a multi-center hospital system in New York City. Annotation was performed by a medical doctor with experience in gynecology. Then, we equally split the notes into train (N = 105) and validation set (N = 150).

#### N2C2 obesity challenge - co-morbidities (PVD, OA and Dep) task

The N2C2 Obesity Challenge dataset [17] consists of 1,237 discharge summaries annotated for obesity and a list of co-morbidities. Annotations are either textual, for information that is explicitly written within notes, or intuitive, reflecting domain expert medical professionals’ reading of the information presented. For our experiments we selected textual annotations as ground truths. We selected a subset of co-morbidities with binary label classes and representing diverse clinical specialties: Osteoarthitis (OA), Depression (Dep), and Peripheral Vascular Disease (PVD). We performed experiments for each of these sub-tasks separately, using annotations for textual information as ground truth. We kept the split into training and test set available from the challenge (PVD: Train N = 506, Test N = 609; OA: Train N = 603, Test N = 502; Dep Train N = 605, Test N = 506). The distribution of the label classes for each task can be found in Appendix B.

#### N2C2 smoking challenge (Smk) task

The N2C2 Smoking Challenge [16] aims at identifying smoking status from clinical notes. The dataset consists of 502 discharge summaries annotated for smoking status with 5 classes: *smoker, current smoker, past smoker, no smoker, unknown*. Because the smoker class had only 3 examples in the test dataset, we merged *current* and *smoker* into a single *current smoker* class. We kept the split into training and test set available from the challenge (Train N = 398, Test N = 104). The distribution of the label classes, for both training and test sets, can be found in Appendix B.

### Prompt design

We adapted the OpenPrompt framework [18] to design our prompt templates and verbalizers. We simulated a situation with minimal prompt engineering, directly translating the task definition and labels into prompt templates and verbalizers (see Table 1).

For all tasks we used the following design principles:

- **Prompt template**: We used prefix-type manual templates of the form:

**<task objective >: [MASK]**

**Fig. 1:**
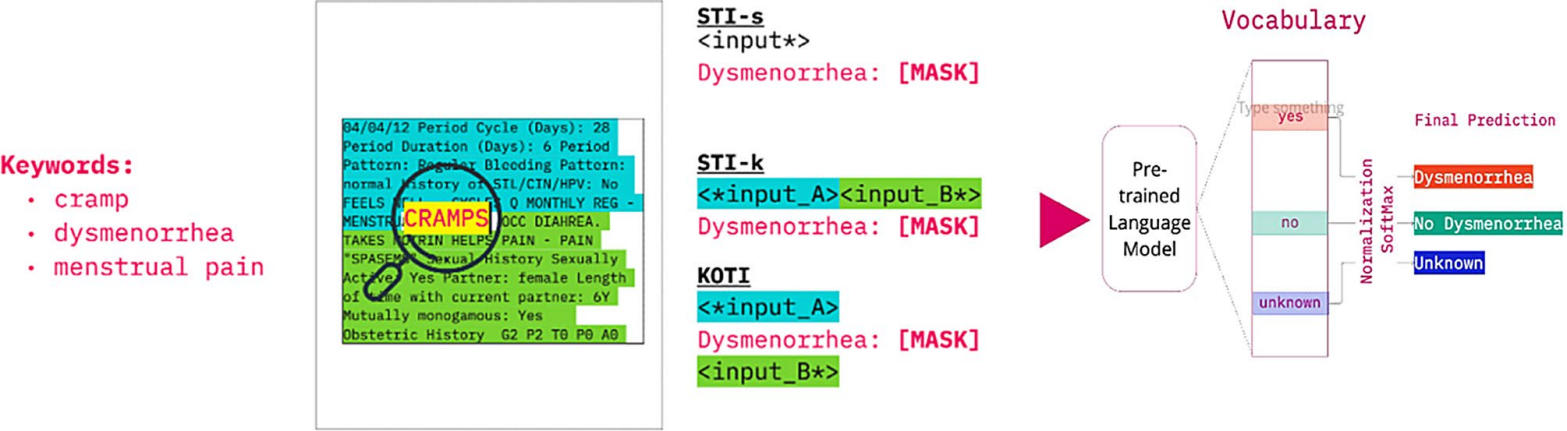
Example of keyword-optimized template insertion for dysmenorrhea task. A set of keywords is used to identify salient regions of clinical notes. The note is split on the keyword-containing sentence and the template is inserted in the vicinity of the salient region. In KOTI, the split input text chunks (<*input_A >, < input_B* >) are then truncated proportionally to their length. STI-k: standard template insertion with keyword chunk, STI-s: standard template insertion with standard chunk

- **Verbalizer**: We used a manual verbalizer as first described by Schick and Schütze [6]. In brief, a set of label words is selected for each class, for example ‘yes’, ‘no’, ‘past’ and ‘unknown’ for label classes ‘smoker’, ‘non smoker’, ‘past smoker’ and ‘unknown’ smoking status. Then, a normalization and softmax step is applied to the label word logits for the model’s prediction of the [MASK] token to identify the predicted class. We selected one single label word per label class (see Table 1 and Fig. 1).

### Template position

#### Keyword-optimized template position (KOTI)

In KOTI, salient regions of the clinical note are identified through keyword-matching, and the prompt template inserted in their vicinity (Fig. 1). For each task, we performed the following steps:

1. **Keyword Definition**: We first built a set of keywords semantically and clinically related to the classes (see Table 1).
2. **Template Insertion**: We then identified sentences within clinical notes and flagged them as containing one or more keywords. We split the clinical note text at the end of the first flagged sentence into two sub-chunks, < text_a > and < text_b >, and inserted the prompt template between them:

     <text_a > < template > < text_b >

When no keyword match was found in the clinical note, the template was appended to the end of the input.

3) **Clinical Note Truncation**: Clinical notes usually contain more tokens than the acceptable input limit of models. When note length exceeded the model’s input limit (i.e. 512 tokens), we performed head truncation, i.e., < trim_text_a >, and tail truncation, i.e., < text_b_trim >, with the number of removed tokens proportional to the sub-chunk’s lengths. The final input resulted as < trim_text_a > < template > < text_b_trim >. In more detail, we first split the clinical note at the end of the identified keyword-containing sentence into two chunks. We then trimmed the chunks so that the chunks together could fit the token number limitation of the pre-trained models (512 tokens for both ClinicalBERT and GatorTron). The number of tokens removed from the two chunks is proportional to the length of the untrimmed chunks. In this way, we ensured that the final model input is centered around the salient sentence identified through the keywords, while respecting the token number limitation of the model.

#### Standard template insertion

We compare KOTI with standard template insertion (STI) at the end of the input text. The truncation method employed in KOTI indirectly selects a “salient” text chunk as the model’s input. We therefore employed and compared two different standard template insertion configurations:

1. **Standard Chunk (STI-s):** The clinical note text is taken as is and tail truncated to fit into the model input (<text_trim >).
2. **Keyword Chunk (STI-k):** The same sub-chunks used in KOTI, i.e., < trim_text_a > and < text_b_trim >, are concatenated and the template text appended at the end of the input.

### Keyword selection

To define the set of keywords for each task, we used a simple method which only included the (1) words and sub-words describing the task (e.g., the OA task included keywords ‘osteo’, ‘arthritis’, ‘osteaoarthritis’); (2) keywords related to the anatomical location for physical disorders (e.g., ‘bone’, ‘joint’, and ‘cartilage’ for OA); (3) general disease categories (e.g., ‘mood’ for the Depression task); and (4) descriptions of the finding (e.g., ‘smoke’ for the smoking task, ‘cramps’ for the dysmenorrhea task). The keyword selection process was performed a-priori, without looking at the training and test datasets, to ensure that results reflected a lower bound performance. Keyword selection was performed by a trained physician and took less than 10 minutes. The distribution of keyword matches across datasets can be found in the supplementary material (Appendix E).

### Decoder models

Our primary focus is on encoder-based models, where the cloze-style prompt allows inserting templates anywhere in the input. However, for comparison, we also evaluated generative models, which rely on an autoregressive (next-token prediction) objective. In this setup, we used the model’s predicted next token after the template and mapped it to a label class via the verbalizer.

Because generative models typically process text in a strictly left-to-right manner, directly inserting a template at a mid-sequence position is not feasible. To address this, we adapted the KOTI method so that we would not discard potentially important information from the tail of the note. Specifically, we prepended the chunk following the detected keyword-containing sentence, denoted as < text_b >, to the front of the initial chunk < text_a > before appending the prompt template. As a result, the final input sequence is organized as < text_b > < text_a > < template >, ensuring the relevant text and the template both remain in the model’s autoregressive context.

### Experimental setup

We compared the performance of two encoder models, GatorTron [19] and ClinicalBERT [20], a decoder-only model, BioGPT [21], as well as a encoder-decoder model, ClinicalT5 [22]. All models were pre-trained, at least partially, on clinical notes. We performed prompt-based model fine-tuning as described in Schick and Schütze [6].

For each task, we compared the performance of KOTI, STI-k, and STI-s in different training settings:

1. **Zero-Shot:** We evaluated out-of-the-box performance of the models on the validation dataset (no fine-tuning).
2. **Few-Shot with Balanced Examples:** We evaluated the performance of the models trained on k = 1, 4, and 10 examples per label class.
3. **Few-Shot with Random Examples:** We evaluated the performance of the models trained on as many examples as in the balanced setting, but randomly sampled to reflect the natural label class distribution. Moreover, we evaluated the performance of the models trained on 50 and 100 training examples.

For encoder models, we also examined how distance from the keyword impacts performance by shifting the prompt insertion 1, 2, 3, 6, or 12 sentences before or after the keyword-containing sentence.

For prompt-based finetuning, we updated all model parameters by minimizing the cross-entropy loss between the verbalizer’s probability output and the true label. We used AdamW as optimizer with weight decay equal 0.01 on all parameters except for layer biases and normalization layers.

We optimized model hyper-parameters (batch size, learning rate, and epoch number) for each combination of task, training configuration, model, and template insertion method via random search. We performed 10 runs for each hyper-parameter combination, where we randomly selected training examples from the training dataset, evaluating performance on the remaining sample. The number of training examples varied from 1 to 100 across the different experimental configurations (see training settings above). The best hyper-parameters were selected based on F1 scores for binary classification tasks and Macro F1 scores, i.e., averaged across label classes, for multiclass classification tasks on the validation sets. Training of the models was performed on one NVIDIA a100 GPU.

For each training setting, we simulated a real-world scenario and randomly sampled k number of training examples from the validation dataset, evaluating the model’s performance on the remaining examples. We repeated this procedure for 10 runs to estimate average precision, recall and F1 scores. We report average F1 scores for multi-class tasks.

### Metrics and statistical analyses

Model performance was assessed in terms of precision, recall, and F1 scores. For multi-class tasks (Dys, Smk), metrics were reported as averages across label classes.

To compare overall performance of KOTI to STI-k and STI-s, we estimated the average F1 score across different tasks. To compare the change in performance across KOTI vs STI-k, KOTI vs STI-s, and STI-k vs STI-s we used average performance for each task and model separately and estimated p-values via paired t-test with significance threshold of 0.05 adjusted via Holm-Bonferroni correction (final threshold of 0.01).

## Results

In the zero-shot setting (Table 2), KOTI generally outperformed both STI-k and STI-s for encoder models, except in the dysmenorrhea task with ClinicalBERT. By contrast, the generative models (BioGPT, ClinicalT5) showed poor zero-shot performance overall and yielded only marginal gains with KOTI.

**Table 2:** Zero-Shot Performance. We report F1 scores for each model and template insertion technique across all five tasks. Dep: Depression, OA: Osteoarthritis, PVD: Peripheral vascular disease, Dys: Dysmenorrhea, Smk: Smoking status

Task	GatroTron			ClinicaBERT			BioGPT			ClinicalT5			
	KOTI	STI-k	STI-s	KOTI	STI-k	STI-s	KOTI	STI-k	STI-s	KOTI	STI-k	STI-s	
**Dep**	**0.354**	0.069	0.164	**0.030**	0.000	0.000	0.000	0.000	0.000	0.000	0.000	0.000	
**OA**	**0.369**	0.021	0.022	**0.045**	0.000	0.000	0.000	0.000	0.000	0.000	0.000	0.000	
**PVD**	**0.305**	0.000	0.029	0.000	0.000	0.000	0.000	0.000	0.000	0.000	0.000	0.000	
**Dys**	**0.414**	0.240	0.156	0.151	**0.154**	**0.154**	0.267	**0.278**	0. 278	0.208	0.208	0.208	
**Smk**	**0.181**	0.152	0.128	**0.164**	0.080	0.068	**0.145**	0.142	0.087	0.067	0.067	0.067	

In the few-shot setting, KOTI continued to provide clear advantages for encoder models (Table 3). Compared to STI-k, KOTI improved macro-F1 scores by an average of 24.12% for GatorTron and 8.35% for ClinicalBERT. Against STI-s, the performance gap widened further, reaching average gains of 77.07% (GatorTron) and 98.92% (ClinicalBERT). Although KOTI consistently enhanced performance across tasks, these improvements were statistically significant only for Depression (Dep), Peripheral Vascular Disease (PVD), and Dysmenorrhea (Dys) with GatorTron, and for Dysmenorrhea with ClinicalBERT. Against STI-s, KOTI achieved significant gains for each encoder-task pair.

**Table 3:** Change in F1 score for KOTI vs STI-k, KOTI vs STIs and STI-k vs STI-s across models and tasks. We report p-values obtained via paired t-test with significance threshold of 0.05 Holm-Bonferroni adjusted. Bold values are significant. Dep: Depression, OA: Osteoarthritis, PVD: Peripheral vascular disease, Dys: Dysmenorrhea, Smk: Smoking status

Task\Model	GatorTron		ClinicalBERT		BioGPT		ClinicalT5	
**KOTI vs STI-k**	$\Delta$**F1**	**p-value**	$\Delta$**F1**	**p-value**	$\Delta$**F1**	**p-value**	$\Delta$**F1**	**p-value**
Dep	**0.1580 (+28.05%)**	**<0.0001**	0.0934 (+17.05%)	0.0274	0.1119 (+26.33%)	0.0167	−**0.1553** (−21.93%)	**<0.0001**
OA	0.0812 (+19.51%)	0.0101	−0.0159 (−3.66%)	0.5813	**0.1009 (+35.19%)**	**0.0004**	**−0.0892 (−17.95%)**	**0.0018**
PVD	**0.1619 (+37.44%)**	**<0.0001**	0.0002 (+0.04%)	0.9949	0.1019 (+30.07%)	0.0125	−0.0660 (−13.05%)	0.0518
Dys	**0.1521 (+26.57%)**	**<0.0001**	**0.0945 (+17.98%)**	**<0.0001**	**0.0510 (+10.98%)**	**0.0089**	**−0.1500 (−21.75%)**	**<0.0001**
Smk	0.0122 (+3.40%)	0.4968	0.0079 (+2.88%)	0.5310	−0.0269 (−10.13%)	0.0178	−0.0232 (−7.75%)	0.0514
*All*	**0.1131 (+24.12%)**	**<0.0001**	**0.0360 (+8.35%)**	**0.0044**	**0.0678 (+19.03)**	**<0.0001**	**−0.0967 (−17.92%)**	**<0.0001**
**KOTI vs STI-s**	$\Delta$**F1**	**p-value**	$\Delta$**F1**	**p-value**	$\Delta$**F1**	**p-value**	$\Delta$**F1**	**p-value**
Dep	**0.4711** (+188.23%)	**<0.0001**	**0.4817 (+302.47%)**	**<0.0001**	**0.3789 (+239.59%)**	**<0.0001**	**0.3896 (+238.32%)**	**<0.0001**
OA	**0.2898 (+139.50%)**	**<0.0001**	**0.2103 (+101.50%)**	**<0.0001**	**0.2284 (+143.53%)**	**<0.0001**	**0.2338 (+134.20%)**	**<0.0001**
PVD	**0.2774 (+87.48%)**	**<0.0001**	**0.2135 (+130.23%)**	**<0.0001**	**0.2591 (+142.77%)**	**<0.0001**	**0.2466 (+128.00%)**	**<0.0001**
Dys	**0.1620 (+28.80%)**	**<0.0001**	**0.2184 (+54.39%)**	**<0.0001**	**0.0974 (+23.27%)**	**<0.0001**	−0.0577 (−9.66%)	0.0190
Smk	**0.0661 (+21.63%)**	**0.0005**	**0.0384 (+15.81%)**	**0.0008**	−0.0076 (−3.07%)	0.5296	−0.0011 (−0.38%)	0.9315
*All*	**0.2533 (+77.07%)**	**<0.0001**	**0.2325 (+98.92%)**	**<0.0001**	**0.1912 (+82.21)**	**<0.0001**	**0.1622 (+57.76%)**	**<0.0001**
**STI-k vs STI-s**	$\Delta$**F1**	**p-value**	$\Delta$**F1**	**p-value**	$\Delta$**F1**	**p-value**	$\Delta$**F1**	**p-value**
Dep	**0.3131 (+125.08%)**	**<0.0001**	**0.3883 (+243.83%)**	**<0.0001**	**0.2670 (+186.80%)**	**<0.0001**	**0.5449 (+333.33%)**	**<0.0001**
OA	**0.2086 (+100.04%)**	**<0.0001**	**0.2262 (+109.16%)**	**<0.0001**	**0.1275 (+80.14%)**	**<0.0001**	**0.3230 (+185.42%)**	**<0.0001**
PVD	**0.1155 (+36.41%)**	**0.0055**	**0.2133 (+130.11%)**	**<0.0001**	**0.1573 (+86.64%)**	**<0.0001**	**0.3126 (+162.24%)**	**<0.0001**
Dys	0.0099 (+1.76%)	0.8011	**0.1239 (+30.86%)**	**<0.0001**	0.0463 (+11.07%)	0.0102	**0.0923 (+25.46%)**	**0.0100**
Smk	**0.0539 (+17.74%)**	**0.0010**	**0.0306 (+12.57%)**	**0.0084**	0.0193 (+07.85%)	0.1150	0.0221 (+7.99%)	0.0682
*All*	**0.1402 (+42.65%)**	**<0.0001**	**0.1965 (+83.60%)**	**<0.0001**	**0.1235 (+53.08%)**	**<0.0001**	**0.2590 (+92.20%)**	**<0.0001**

In contrast, results for generative models (BioGPT, ClinicalT5) were more variable. BioGPT demonstrated overall improvements when comparing KOTI to STI-k (+19.03%), whereas ClinicalT5 performance dropped (−17.92%). However, compared with STI-s, KOTI yielded a more broadly positive effect, albeit with some task-specific inconsistencies. Across all models, using a keyword-focused chunk (STI-k) was still preferable to naive tail-truncation (STI-s), although the gains were not universally significant on a per-task basis.

**Fig. 2:**
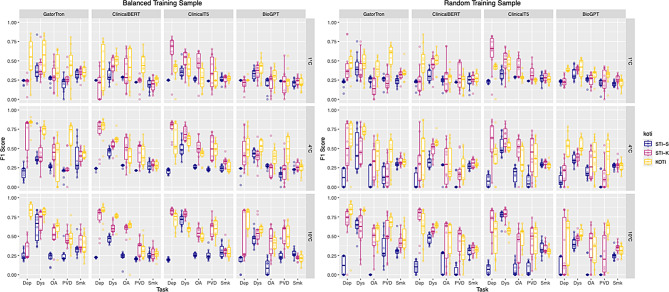
Performance Across Tasks. F1 and Macro-F1 scores across the tasks reported. C = number of classes

Figure 2 reports the performance of each template insertion methods across tasks, model and training strategy. The benefit of using KOTI over STI-k is highest with lower number of training examples, and is variable across tasks.

**Fig. 3:**
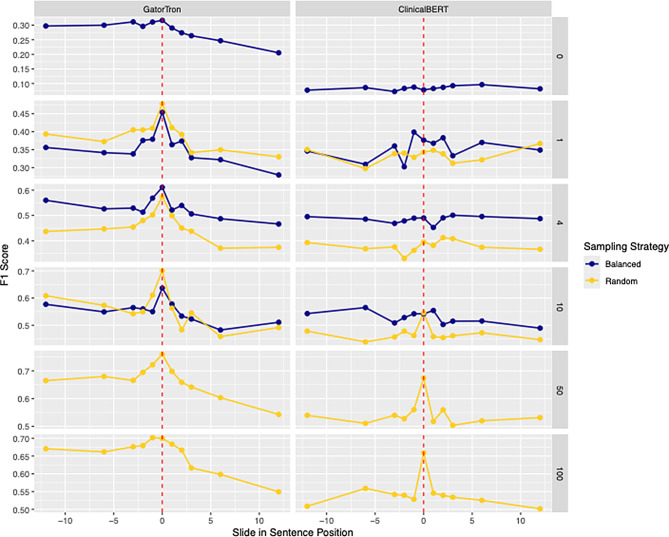
Average performance across tasks when varying the template position with respect to the keyword-containing sentence. Dashed red lines represent template insertion at the end of the first keyword-containing sentence

Figure 3 illustrates how shifting the template insertion relative to the keyword sentence affects model performance. For encoder models, F1 scores peaked when the prompt was placed at or near the keyword-containing sentence, then declined more sharply when the distance was increased. This trend was especially pronounced for GatorTron and became more evident as the number of training examples grew.

Table 3 presents the best performance for each combination of task, template insertion technique, and pre-trained model. Overall, GatorTron was the top performer, achieving the highest F1 scores across all tasks. Notably, KOTI provided the best results for every task except PVD when used with encoder models—succeeding in 7 out of 10 encoder-based scenarios—whereas with decoder models, KOTI outperforms STI-k only 2 out of 10 times. Complete results for all experimental configurations are available in Appendix B.

**Table 4:** Best Performing Models. The F1 scores for the best performing combination of training samples and sampling strategy across template insertion technique are given. Bold values highlight best template configurations for each model and task; green bold values highlight best overall model for each task. N: number of training examples. C: number of label classes. Dep: Depression, OA: Osteoarthritis, PVD: Peripheral vascular disease, Dys: Dysmenorrhea, Smk: Smoking status

Model	GatorTron				ClinicalBERT				BioGPT				ClinicalT5			
Task	Template	F1	N	Sampling	Template	F1	N	Sampling	Template	F1	N	Sampling	Template	F1	N	Sampling
**Dep**	**KOTI** STI-k STI-s	**0.919** 0.910 0.534	**100** 100 100	**Random** Random Random	**KOTI** STI-k STI-s	**0.885** 0.812 0.247	100 100 1*C	Random Random Balanced	KOTI **STI-k** STI-s	0.800 **0.807** 0.246	100 100 1	Random Random Balanced	KOTI **STI-k** STI-s	0.812 **0.850** 0.249	100 100 1	Random Random Balanced
**OA**	**KOTI** STI-k STI-s	**0.693** 0.640 0.376	**100** 100 100	**Random** Random Random	KOTI **STI-k** STI-s	0.568 **0.598** 0.283	10*C 10*C 1*C	Balanced Balanced Balanced	**KOTI** STI-k STI-s	**0.566** 0.435 0.268	50 10 4	Random Balanced Balanced	KOTI **STI-k** STI-s	0.538 **0.638** 0.288	50 10 1	Random Random Random
**PVD**	KOTI **STI-k** STI-s	0.815 **0.840** 0.641	100 **100** 100	Random **Random** Random	KOTI **STI-k** STI-s	0.599 **0.620** 0.275	100 100 100	Random Random Random	**KOTI** STI-k STI-s	**0.664** 0.632 0.413	100 100 100	Random Random Random	KOTI **STI-k** STI-s	0.636 **0.746** 0.327	100 100 100	Random Random Random
**Dys**	**KOTI** STI-k STI-s	**0.860** 0.846 0.843	**100** 100 100	**Random** Random Random	**KOTI** STI-k STI-s	**0.750** 0.612 0.485	100 10 100	Random Balanced Random	KOTI **STI-k** STI-s	0.588 **0.620** 0.573	50 100 100	Random Random Random	KOTI STI-k **STI-s**	0.616 0.840 **0.849**	100 100 100	Random Random Random
**Smk**	**KOTI** STI-k STI-s	**0.471** 0.424 0.367	**10** 10 4*C	**Random** Random Balanced	**KOTI** STI-k STI-s	**0.346** 0.335 0.298	50 50 10	Random Random Random	KOTI **STI-k** STI-s	0.318 **0.347** 0.296	10 10 50	Random Random Random	KOTI **STI-k** STI-s	0.323 **0.347** 0.343	50 10 10	Random Random Random

## Discussion

Our work highlights the importance of template position in prompt-based learning, a factor often overlooked in prior research. In zero-shot classification of clinical notes, the KOTI approach outperformed both STI-k and STI-s for encoder models, suggesting that strategic prompt placement can compensate for limited training data. We also observed a notable benefit of KOTI in few-shot settings, compared to STI-k, confirming that template placement alone—using the same input text chunk—drives the performance gains. Furthermore, our analysis of how template distance from keyword-containing sentences influences performance supports the idea that positioning prompts near salient text is critical for optimal results. This advantage, however, varies across tasks, aligning with previous evidence that prompt-related factors can be highly context- or domain-dependent (Table 4).

Generative (decoder-based) models, such as BioGPT and ClinicalT5, exhibited more variable outcomes with KOTI. Although BioGPT benefited from KOTI’s keyword-focused strategy, these gains were inconsistent across tasks. Meanwhile, ClinicalT5—a larger encoder-decoder model—often experienced diminished performance when the template was inserted via KOTI. A plausible explanation is that prepending the text chunk after the keyword disrupts the normal semantic flow, causing difficulties in the encoder-decoder pipeline. Specifically, the encoder processes an input sequence where logical order has been inverted, which can degrade the richness of the encoded representation prior to decoding.

By contrast, GatorTron (an encoder-only model) surpassed other models despite having fewer parameters than ClinicalT5 (~350 million vs ~ 740 million parameters). One possible reason is that GatorTron was extensively pre-trained on a large and diverse corpus of clinical notes, giving it stronger domain adaptation that synergizes well with KOTI. Because GatorTron encodes text bidirectionally, without the additional decoding step, inserting the template at or near clinically salient sentences imposes less disruption to the input’s overall structure. This architecture is likely to also contribute to GatorTron’s stable performance across tasks and explain why KOTI consistently drives more pronounced improvements in encoder-based models compared to decoder-only or encoder-decoder setups.

We also observed that naïve truncation (STI-s), which can discard vital segments of text, consistently lagged behind with both KOTI and STI-k. This drawback was particularly pronounced with ClinicalBERT, where performance plateaued even as the training set expanded. Other approaches, such as the one proposed by Sivarajkumar and Wang [10], circumvent truncation by splitting clinical notes into multiple chunks and running separate inference on each. However, this method substantially increases computational overhead—on the N2C2 obesity dataset, for instance, about 98% of notes exceed the 512-token limit for our models and would require around three passes each (see Appendix D). In contrast, KOTI delivered competitive accuracy with a single pass per note, providing a more efficient solution that preserves crucial context around keyword-identified regions.

Finally, KOTI’s impact on performance was most pronounced when the amount of training data was minimal, reflecting its value in resource-scarce environments. As datasets grow larger, the advantage of precise prompt placement diminishes but does not entirely disappear.

### Limitations

We report several limitations in our work. Firstly, our method required the engineering of effective keywords. While we showed promising results using *a priori* keywords selection with limited engineering, we achieved the best results on the dysmenorrhea task for which keywords were selected by a domain expert. Second, we did not leverage prompting techniques (e.g., in-context learning) that are often employed with large language models. Instead, we maintained a comparable setup across encoder and decoder models to enable direct comparisons. Consequently, although we observed that GatorTron outperformed decoder-based ClinicalT5 despite having fewer parameters, this finding may not hold if more sophisticated prompting strategies are used. Finally, keyword matching requires some degree of computational efforts for note processing and text pattern matching. Further studies should assess the benefits of KOTI over full note processing such as in Sivarajkumar and Wang [9].

## Conclusion

Our findings underscore how template positioning can substantially influence the performance and efficiency of clinical note classification. The KOTI method leverages simple keyword matching to place prompts in salient regions and reduce the risk of discarding crucial text. Overall, these results highlight that the value of KOTI grows as training data become scarcer. Moreover, we show that encoder-based models—particularly GatorTron—reap the most significant benefits as they are naturally enabling the positioning of the template arbitrarily within the input text.

## Electronic supplementary material

Below is the link to the electronic supplementary material.

Supplementary Material 1

## Data Availability

N2C2 Obesity and Smoking Challenge data is available upon registration and approval at https://portal.dbmi.hms.harvard.edu/projects/n2c2-nlp/. Mount Sinai data used to develop the dysmenorrhea task is only available upon request to the authors and following approval by competent offices within Mount Sinai. The code used to generate the results as well as implement keyword optimization (KOTI) is available at https://github.com/eugenial/koti/. We acknowledge the support from the computational and data resources and staff expertise provided by Scientific Computing and Data at the Icahn School of Medicine at Mount Sinai and supported by the Clinical and Translational Science Awards (CTSA) grant UL1TR004419 from the National Center for Advancing Translational Sciences.
