# Supplementary material for: Keyword-optimized template insertion for clinical note classification via prompt-based learning
Source: BMC Med Inform Decis Mak. 2025 Jul 3;25:247. doi: 10.1186/s12911-025-03071-y (PMC12224782; doi:10.1186/s12911-025-03071-y)
Supplement: Supplementary file 1 — Supplementary Material 1 [file 12911_2025_3071_MOESM1_ESM.docx]

**SUPPLEMENTARY**

**Appendix A: Hyperparameter running and training**

Hyper-parameter where optimized via 10 trials of random search over the following hyper-parameter space:

- Learning Rate over a random uniform distribution on [1e-4; 1e-7]
- Batch size over the set {1,2,4}
- Number of epochs over the set {1,2,3,4,5,6,7,8,9,10}

The optimal hyper-parameters where selected based of F1 scores for binary tasks and MacroF1 scores for multiclass tasks.

Training of the models was performed on NVIDIA h100 GPUs.

**Appendix B: Class Distribution Across Tasks**

| **Task** | **Class** | **Train** | **Validation** |
| --- | --- | --- | --- |
| Dys | Yes  No | 34  52 | 37  45 |
|  | Unknown | 64 | 68 |
| Dep | Yes  Unknown | 86  519 | 72  434 |
| PVD | Yes  Unknown | 83  526 | 65  443 |
| OA | Yes  Unknown | 89  514 | 86  416 |
| Smk | Current Smoker  Past Smoker  No Smoker | 44  36  66 | 14  11  16 |
|  | Unknown | 252 | 63 |

Table 4: Class distribution for different tasks.

**Appendix C: Additional Results**

We report the average F1 scores across each experimental setting separately for each task in Table C1-C5.

**Depression Task with Balanced Training Examples**

| N | Template | GatorTron | 95%LCI | 95%UCI | ClinicalBERT | 95%LCI | 95%UCI | BioGPT | 95%LCI | 95%UCI | ClinicalT5 | 95%LCI | 95%UCI |
| --- | --- | --- | --- | --- | --- | --- | --- | --- | --- | --- | --- | --- | --- |
| 0 | KOTI | 0.354 | 0.354 | 0.354 | 0.0304 | 0.0304 | 0.0304 | 0 | 0 | 0 | 0 | 0 | 0 |
| 0 | STI-K | 0.0694 | 0.0694 | 0.0694 | 0 | 0 | 0 | 0 | 0 | 0 | 0 | 0 | 0 |
| 0 | STI-S | 0.1642 | 0.1642 | 0.1642 | 0 | 0 | 0 | 0 | 0 | 0 | 0 | 0 | 0 |
| 1 | KOTI | 0.5281 | 0.0548 | 0.8552 | 0.4077 | 0.0541 | 0.7914 | 0.2553 | 0.247 | 0.3256 | 0.3739 | 0 | 0.497 |
| 1 | STI-K | 0.227 | 0.0278 | 0.3445 | 0.1886 | 0 | 0.4815 | 0.1999 | 0.0278 | 0.2953 | 0.6066 | 0.0615 | 0.8153 |
| 1 | STI-S | 0.2327 | 0.1757 | 0.2491 | 0.247 | 0.247 | 0.247 | 0.2463 | 0.2434 | 0.247 | 0.2485 | 0.2442 | 0.2586 |
| 4 | KOTI | 0.8232 | 0.6083 | 0.9078 | 0.7558 | 0.3976 | 0.8841 | 0.511 | 0.16 | 0.8489 | 0.5933 | 0.2982 | 0.8516 |
| 4 | STI-K | 0.6761 | 0.2524 | 0.8442 | 0.705 | 0.3018 | 0.8516 | 0.4533 | 0.1805 | 0.8158 | 0.767 | 0.549 | 0.8571 |
| 4 | STI-S | 0.1881 | 0.055 | 0.3309 | 0.2354 | 0.1905 | 0.2509 | 0.2097 | 0.0253 | 0.2443 | 0.2067 | 0.1493 | 0.2425 |
| 10 | KOTI | 0.8339 | 0.7179 | 0.9375 | 0.8111 | 0.5849 | 0.9231 | 0.728 | 0.4976 | 0.8507 | 0.7094 | 0.4938 | 0.8369 |
| 10 | STI-K | 0.3238 | 0.2263 | 0.552 | 0.7334 | 0.4096 | 0.8511 | 0.4769 | 0.1797 | 0.8451 | 0.823 | 0.7746 | 0.8722 |
| 10 | STI-S | 0.25 | 0.1721 | 0.3681 | 0.2244 | 0.2125 | 0.2311 | 0.1708 | 0 | 0.2313 | 0.1968 | 0.1675 | 0.2284 |

**Depression Task with Random Training Examples**

| N | Template | GatorTron | 95%LCI | 95%UCI | ClinicalBERT | 95%LCI | 95%UCI | BioGPT | 95%LCI | 95%UCI | ClinicalT5 | 95%LCI | 95%UCI |
| --- | --- | --- | --- | --- | --- | --- | --- | --- | --- | --- | --- | --- | --- |
| 1 | KOTI | 0.4566 | 0.1687 | 0.6333 | 0.4685 | 0.1266 | 0.8428 | 0.3872 | 0.3636 | 0.4385 | 0.3721 | 0 | 0.4674 |
| 1 | STI-K | 0.4385 | 0.247 | 0.8481 | 0.2535 | 0.0548 | 0.6829 | 0.2325 | 0.1532 | 0.3438 | 0.6013 | 0.0609 | 0.8227 |
| 1 | STI-S | 0.2106 | 0.0741 | 0.2624 | 0.2141 | 0.0862 | 0.2533 | 0.2261 | 0.1796 | 0.2507 | 0.2299 | 0.197 | 0.2504 |
| 4 | KOTI | 0.616 | 0 | 0.8462 | 0.4208 | 0 | 0.8725 | 0.4582 | 0 | 0.7852 | 0.383 | 0 | 0.8382 |
| 4 | STI-K | 0.5213 | 0.0811 | 0.8533 | 0.3166 | 0 | 0.8462 | 0.1939 | 0 | 0.4259 | 0.5955 | 0.0526 | 0.875 |
| 4 | STI-S | 0.1236 | 0 | 0.2491 | 0.0735 | 0 | 0.246 | 0.0886 | 0 | 0.256 | 0.0857 | 0 | 0.2092 |
| 10 | KOTI | 0.7643 | 0.0556 | 0.9565 | 0.6076 | 0.1772 | 0.8442 | 0.4372 | 0 | 0.8378 | 0.5058 | 0.0556 | 0.7941 |
| 10 | STI-K | 0.6671 | 0 | 0.848 | 0.7144 | 0.2824 | 0.8592 | 0.3215 | 0 | 0.8456 | 0.663 | 0.2716 | 0.8592 |
| 10 | STI-S | 0.1225 | 0 | 0.2482 | 0.1007 | 0 | 0.2051 | 0.0961 | 0 | 0.245 | 0.0766 | 0 | 0.1901 |
| 50 | KOTI | 0.8664 | 0.7358 | 0.9466 | 0.8321 | 0.7586 | 0.8872 | 0.7727 | 0.6263 | 0.8525 | 0.7301 | 0.5684 | 0.843 |
| 50 | STI-K | 0.793 | 0.6531 | 0.9474 | 0.712 | 0.4444 | 0.8472 | 0.7581 | 0.566 | 0.8527 | 0.8312 | 0.5979 | 0.9209 |
| 50 | STI-S | 0.3494 | 0.0488 | 0.6087 | 0.1043 | 0 | 0.2222 | 0.1082 | 0 | 0.2941 | 0.0761 | 0 | 0.2276 |
| 100 | KOTI | 0.9194 | 0.8704 | 0.9533 | 0.8853 | 0.8519 | 0.9123 | 0.8004 | 0.7312 | 0.864 | 0.812 | 0.7965 | 0.8257 |
| 100 | STI-K | 0.9096 | 0.8545 | 0.9541 | 0.8121 | 0.7253 | 0.873 | 0.8072 | 0.6869 | 0.85 | 0.85 | 0.8119 | 0.896 |
| 100 | STI-S | 0.5339 | 0.3918 | 0.6214 | 0.0907 | 0 | 0.186 | 0.1351 | 0.0328 | 0.2687 | 0.2037 | 0 | 0.5287 |

**Dysmenorrhea Task with Balanced Training Examples**

| N | Template | GatorTron | 95%LCI | 95%UCI | ClinicalBERT | 95%LCI | 95%UCI | BioGPT | 95%LCI | 95%UCI | ClinicalT5 | 95%LCI | 95%UCI |
| --- | --- | --- | --- | --- | --- | --- | --- | --- | --- | --- | --- | --- | --- |
| 0 | KOTI | 0 | KOTI | 0.3728 | 0.3728 | 0.3728 | 0.1509 | 0.1509 | 0.1509 | 0.2668 | 0.2668 | 0.2668 | 0.208 |
| 0 | STI-K | 0 | STI-K | 0.2161 | 0.2161 | 0.2161 | 0.1538 | 0.1538 | 0.1538 | 0.2776 | 0.2776 | 0.2776 | 0.208 |
| 0 | STI-S | 0 | STI-S | 0.1554 | 0.1554 | 0.1554 | 0.1538 | 0.1538 | 0.1538 | 0.2722 | 0.2722 | 0.2722 | 0.208 |
| 1 | KOTI | 1 | KOTI | 0.5698 | 0.2941 | 0.8589 | 0.4896 | 0.1936 | 0.6615 | 0.4013 | 0.1788 | 0.6046 | 0.4043 |
| 1 | STI-K | 1 | STI-K | 0.415 | 0.2198 | 0.8108 | 0.4312 | 0.2405 | 0.5689 | 0.3546 | 0.1745 | 0.4823 | 0.5107 |
| 1 | STI-S | 1 | STI-S | 0.3964 | 0.239 | 0.8395 | 0.3061 | 0.1627 | 0.4785 | 0.3283 | 0.2407 | 0.4413 | 0.3684 |
| 4 | KOTI | 4 | KOTI | 0.7425 | 0.6264 | 0.8579 | 0.6121 | 0.3986 | 0.7958 | 0.5127 | 0.2462 | 0.6263 | 0.6046 |
| 4 | STI-K | 4 | STI-K | 0.4384 | 0.1287 | 0.7767 | 0.5339 | 0.3749 | 0.6126 | 0.3995 | 0.1469 | 0.6533 | 0.698 |
| 4 | STI-S | 4 | STI-S | 0.4065 | 0.3029 | 0.8257 | 0.4561 | 0.37 | 0.5352 | 0.4322 | 0.3201 | 0.5043 | 0.4988 |
| 10 | KOTI | 10 | KOTI | 0.8069 | 0.6792 | 0.8657 | 0.7408 | 0.5259 | 0.794 | 0.5861 | 0.5014 | 0.6516 | 0.5955 |
| 10 | STI-K | 10 | STI-K | 0.6962 | 0.4624 | 0.8387 | 0.6116 | 0.516 | 0.7398 | 0.5572 | 0.398 | 0.8239 | 0.7865 |
| 10 | STI-S | 10 | STI-S | 0.6209 | 0.3584 | 0.8405 | 0.4642 | 0.3866 | 0.5326 | 0.4721 | 0.3228 | 0.6599 | 0.707 |

**Dysmenorrhea Task with Random Training Examples**

| N | Template | GatorTron | 95%LCI | 95%UCI | ClinicalBERT | 95%LCI | 95%UCI | BioGPT | 95%LCI | 95%UCI | ClinicalT5 | 95%LCI | 95%UCI |
| --- | --- | --- | --- | --- | --- | --- | --- | --- | --- | --- | --- | --- | --- |
| 1 | KOTI | 1 | KOTI | 0.4494 | 0.1461 | 0.6477 | 0.4885 | 0.2885 | 0.6854 | 0.3966 | 0.1713 | 0.5388 | 0.4496 |
| 1 | STI-K | 1 | STI-K | 0.4022 | 0.2298 | 0.7497 | 0.4387 | 0.1602 | 0.6143 | 0.3907 | 0.329 | 0.443 | 0.4927 |
| 1 | STI-S | 1 | STI-S | 0.4208 | 0.2399 | 0.7521 | 0.3123 | 0.2091 | 0.4472 | 0.3317 | 0.2211 | 0.4726 | 0.3263 |
| 4 | KOTI | 4 | KOTI | 0.7267 | 0.6206 | 0.8223 | 0.589 | 0.5395 | 0.7621 | 0.5405 | 0.4194 | 0.695 | 0.5286 |
| 4 | STI-K | 4 | STI-K | 0.4916 | 0.2641 | 0.847 | 0.4921 | 0.2861 | 0.6249 | 0.3879 | 0.2734 | 0.5011 | 0.6409 |
| 4 | STI-S | 4 | STI-S | 0.4639 | 0.1812 | 0.8429 | 0.3232 | 0.2279 | 0.4656 | 0.3546 | 0.2302 | 0.4577 | 0.5254 |
| 10 | KOTI | 10 | KOTI | 0.833 | 0.7851 | 0.8723 | 0.6368 | 0.5541 | 0.6851 | 0.5399 | 0.449 | 0.6497 | 0.5652 |
| 10 | STI-K | 10 | STI-K | 0.5802 | 0.1333 | 0.8474 | 0.5765 | 0.4948 | 0.6581 | 0.4758 | 0.3773 | 0.6012 | 0.7727 |
| 10 | STI-S | 10 | STI-S | 0.6275 | 0.3698 | 0.7344 | 0.4651 | 0.3655 | 0.5486 | 0.4037 | 0.3039 | 0.557 | 0.7578 |
| 50 | KOTI | 50 | KOTI | 0.843 | 0.6898 | 0.8778 | 0.7 | 0.5056 | 0.827 | 0.588 | 0.4884 | 0.6835 | 0.586 |
| 50 | STI-K | 50 | STI-K | 0.7491 | 0.6122 | 0.8713 | 0.5577 | 0.2257 | 0.6557 | 0.5515 | 0.2005 | 0.7768 | 0.8238 |
| 50 | STI-S | 50 | STI-S | 0.7592 | 0.6574 | 0.8281 | 0.4248 | 0.3129 | 0.4967 | 0.466 | 0.2716 | 0.6806 | 0.7838 |
| 100 | KOTI | 100 | KOTI | 0.8604 | 0.7817 | 0.92 | 0.7489 | 0.6435 | 0.8117 | 0.5861 | 0.4608 | 0.6925 | 0.6156 |
| 100 | STI-K | 100 | STI-K | 0.8426 | 0.7158 | 0.9306 | 0.5987 | 0.5107 | 0.7304 | 0.6196 | 0.3741 | 0.8186 | 0.8392 |
| 100 | STI-S | 100 | STI-S | 0.8457 | 0.7372 | 0.921 | 0.485 | 0.3112 | 0.6863 | 0.5732 | 0.4159 | 0.7309 | 0.8491 |

**Osteoarthritis Task with Balanced Training Examples**

| N | Template | GatorTron | 95%LCI | 95%UCI | ClinicalBERT | 95%LCI | 95%UCI | BioGPT | 95%LCI | 95%UCI | ClinicalT5 | 95%LCI | 95%UCI |
| --- | --- | --- | --- | --- | --- | --- | --- | --- | --- | --- | --- | --- | --- |
| 0 | KOTI | KOTI | 0.3688 | 0.3688 | 0.3688 | 0.0451 | 0.0451 | 0.0451 | 0 | 0 | 0 | 0 | 0 |
| 0 | STI-K | STI-K | 0.0206 | 0.0206 | 0.0206 | 0 | 0 | 0 | 0 | 0 | 0 | 0 | 0 |
| 0 | STI-S | STI-S | 0.0217 | 0.0217 | 0.0217 | 0 | 0 | 0 | 0 | 0 | 0 | 0 | 0 |
| 1 | KOTI | KOTI | 0.3873 | 0.0455 | 0.657 | 0.4179 | 0.0479 | 0.6911 | 0.3008 | 0.0841 | 0.4641 | 0.2739 | 0.0632 |
| 1 | STI-K | STI-K | 0.3066 | 0.0743 | 0.5854 | 0.3487 | 0.065 | 0.6887 | 0.2243 | 0 | 0.4113 | 0.4402 | 0.0457 |
| 1 | STI-S | STI-S | 0.2604 | 0.1493 | 0.2962 | 0.2826 | 0.2487 | 0.2926 | 0.194 | 0 | 0.2692 | 0.2656 | 0.2263 |
| 4 | KOTI | KOTI | 0.3758 | 0 | 0.7108 | 0.4152 | 0.185 | 0.6796 | 0.3847 | 0.1702 | 0.5594 | 0.4103 | 0.2463 |
| 4 | STI-K | STI-K | 0.445 | 0.1397 | 0.6425 | 0.4881 | 0.1587 | 0.6863 | 0.2525 | 0.1132 | 0.6667 | 0.5093 | 0.2883 |
| 4 | STI-S | STI-S | 0.2588 | 0.1782 | 0.3106 | 0.2777 | 0.2378 | 0.2852 | 0.2681 | 0.2083 | 0.2959 | 0.2613 | 0.238 |
| 10 | KOTI | KOTI | 0.5846 | 0.1898 | 0.716 | 0.5677 | 0 | 0.6957 | 0.402 | 0.2418 | 0.5517 | 0.4915 | 0.4297 |
| 10 | STI-K | STI-K | 0.5098 | 0.1966 | 0.6531 | 0.5979 | 0.4536 | 0.6484 | 0.4353 | 0.1364 | 0.6561 | 0.5479 | 0.4298 |
| 10 | STI-S | STI-S | 0.2198 | 0.0877 | 0.2869 | 0.2304 | 0.0417 | 0.2958 | 0.0987 | 0 | 0.2547 | 0.2558 | 0.216 |

**Osteoarthritis Task with Random Training Examples**

| N | Template | GatorTron | 95%LCI | 95%UCI | ClinicalBERT | 95%LCI | 95%UCI | BioGPT | 95%LCI | 95%UCI | ClinicalT5 | 95%LCI | 95%UCI |
| --- | --- | --- | --- | --- | --- | --- | --- | --- | --- | --- | --- | --- | --- |
| 1 | KOTI | 1 | KOTI | 0.3912 | 0.1628 | 0.5727 | 0.278 | 0.0962 | 0.4845 | 0.3158 | 0.1356 | 0.4162 | 0.2888 |
| 1 | STI-K | 1 | STI-K | 0.1619 | 0 | 0.4 | 0.3018 | 0.0932 | 0.689 | 0.2577 | 0.1111 | 0.3485 | 0.4206 |
| 1 | STI-S | 1 | STI-S | 0.2528 | 0.1549 | 0.3059 | 0.2352 | 0.08 | 0.2986 | 0.2512 | 0.126 | 0.3106 | 0.2877 |
| 4 | KOTI | 4 | KOTI | 0.4091 | 0 | 0.6829 | 0.308 | 0.0889 | 0.6798 | 0.3658 | 0 | 0.6866 | 0.3278 |
| 4 | STI-K | 4 | STI-K | 0.3056 | 0.0227 | 0.6667 | 0.2987 | 0 | 0.6633 | 0.2914 | 0.0851 | 0.5475 | 0.4774 |
| 4 | STI-S | 4 | STI-S | 0.1155 | 0 | 0.2931 | 0.2594 | 0 | 0.2931 | 0.1915 | 0.0825 | 0.2805 | 0.1663 |
| 10 | KOTI | 10 | KOTI | 0.5352 | 0.2857 | 0.6707 | 0.4457 | 0 | 0.6667 | 0.3208 | 0 | 0.5966 | 0.465 |
| 10 | STI-K | 10 | STI-K | 0.4613 | 0.068 | 0.6458 | 0.4629 | 0 | 0.68 | 0.3864 | 0 | 0.6837 | 0.3614 |
| 10 | STI-S | 10 | STI-S | 0 | 0 | 0 | 0.172 | 0 | 0.2963 | 0.0571 | 0 | 0.2872 | 0.0723 |
| 50 | KOTI | 50 | KOTI | 0.6174 | 0.3505 | 0.7133 | 0.3854 | 0 | 0.7018 | 0.5657 | 0.0519 | 0.7293 | 0.5097 |
| 50 | STI-K | 50 | STI-K | 0.5399 | 0.1739 | 0.7556 | 0.527 | 0.1304 | 0.6821 | 0.3835 | 0 | 0.596 | 0.6383 |
| 50 | STI-S | 50 | STI-S | 0.1965 | 0.0208 | 0.437 | 0.1098 | 0.0247 | 0.2151 | 0.142 | 0 | 0.2873 | 0.0421 |
| 100 | KOTI | 100 | KOTI | 0.6928 | 0.5686 | 0.768 | 0.5595 | 0.3925 | 0.6479 | 0.4832 | 0.1481 | 0.637 | 0.5379 |
| 100 | STI-K | 100 | STI-K | 0.6401 | 0.0571 | 0.875 | 0.4855 | 0.0274 | 0.6752 | 0.0908 | 0 | 0.6835 | 0.6326 |
| 100 | STI-S | 100 | STI-S | 0.3768 | 0.0789 | 0.6667 | 0.1113 | 0.046 | 0.2016 | 0.0862 | 0 | 0.2996 | 0.0601 |

**Peripheral Vascular Task Disease with Balanced Training Examples**

| N | Template | GatorTron | 95%LCI | 95%UCI | ClinicalBERT | 95%LCI | 95%UCI | BioGPT | 95%LCI | 95%UCI | ClinicalT5 | 95%LCI | 95%UCI |
| --- | --- | --- | --- | --- | --- | --- | --- | --- | --- | --- | --- | --- | --- |
| 0 | KOTI | KOTI | 0.3047 | 0.3047 | 0.3047 | 0 | 0 | 0 | 0 | 0 | 0 | 0 | 0 |
| 0 | STI-K | STI-K | 0 | 0 | 0 | 0 | 0 | 0 | 0 | 0 | 0 | 0 | 0 |
| 0 | STI-S | STI-S | 0.0294 | 0.0294 | 0.0294 | 0 | 0 | 0 | 0 | 0 | 0 | 0 | 0 |
| 1 | KOTI | KOTI | 0.4258 | 0 | 0.8062 | 0.4413 | 0.1266 | 0.713 | 0.2561 | 0.101 | 0.5649 | 0.2727 | 0.1484 |
| 1 | STI-K | STI-K | 0.2934 | 0.1923 | 0.5514 | 0.2166 | 0 | 0.3355 | 0.2166 | 0 | 0.5699 | 0.3494 | 0.0622 |
| 1 | STI-S | STI-S | 0.203 | 0 | 0.3524 | 0.2218 | 0.2218 | 0.2218 | 0.2383 | 0.2358 | 0.2432 | 0.2383 | 0.238 |
| 4 | KOTI | KOTI | 0.6625 | 0.2873 | 0.8288 | 0.4162 | 0.24 | 0.6857 | 0.4656 | 0.0941 | 0.6957 | 0.4835 | 0.2579 |
| 4 | STI-K | STI-K | 0.2642 | 0 | 0.5405 | 0.4486 | 0.2086 | 0.5857 | 0.2365 | 0 | 0.5652 | 0.5099 | 0.3297 |
| 4 | STI-S | STI-S | 0.1927 | 0.0312 | 0.25 | 0.2147 | 0.2147 | 0.2147 | 0.1567 | 0 | 0.2532 | 0.2271 | 0.2013 |
| 10 | KOTI | KOTI | 0.4816 | 0.2449 | 0.7736 | 0.3341 | 0.2038 | 0.5753 | 0.5108 | 0.3517 | 0.7176 | 0.5962 | 0.3774 |
| 10 | STI-K | STI-K | 0.4722 | 0.3406 | 0.6515 | 0.3506 | 0.1858 | 0.5244 | 0.5148 | 0.2222 | 0.7051 | 0.6045 | 0.4569 |
| 10 | STI-S | STI-S | 0.2497 | 0.1818 | 0.5033 | 0.2047 | 0.1702 | 0.2295 | 0.2534 | 0.1605 | 0.4713 | 0.2573 | 0.1801 |

**Peripheral Vascular Disease Task with Random Training Examples**

| N | Template | GatorTron | 95%LCI | 95%UCI | ClinicalBERT | 95%LCI | 95%UCI | BioGPT | 95%LCI | 95%UCI | ClinicalT5 | 95%LCI | 95%UCI |
| --- | --- | --- | --- | --- | --- | --- | --- | --- | --- | --- | --- | --- | --- |
| 1 | KOTI | 1 | KOTI | 0.5475 | 0.244 | 0.7377 | 0.2231 | 0.0435 | 0.4 | 0.302 | 0.0498 | 0.4195 | 0.2475 |
| 1 | STI-K | 1 | STI-K | 0.2483 | 0.0857 | 0.4551 | 0.305 | 0.0714 | 0.5111 | 0.2441 | 0 | 0.4286 | 0.3321 |
| 1 | STI-S | 1 | STI-S | 0.2558 | 0.092 | 0.3313 | 0.2236 | 0.2182 | 0.2423 | 0.199 | 0.0336 | 0.2596 | 0.238 |
| 4 | KOTI | 4 | KOTI | 0.4234 | 0.0312 | 0.6863 | 0.2581 | 0.0312 | 0.5495 | 0.3941 | 0 | 0.6901 | 0.3489 |
| 4 | STI-K | 4 | STI-K | 0.1821 | 0 | 0.4848 | 0.2024 | 0 | 0.5273 | 0.1584 | 0 | 0.5409 | 0.4101 |
| 4 | STI-S | 4 | STI-S | 0.152 | 0 | 0.3117 | 0.0441 | 0 | 0.2238 | 0.0477 | 0 | 0.2398 | 0.0809 |
| 10 | KOTI | 10 | KOTI | 0.6595 | 0.4444 | 0.7961 | 0.347 | 0 | 0.5153 | 0.56 | 0.0241 | 0.7092 | 0.4467 |
| 10 | STI-K | 10 | STI-K | 0.5128 | 0.129 | 0.8235 | 0.4286 | 0 | 0.6115 | 0.2715 | 0 | 0.6667 | 0.4434 |
| 10 | STI-S | 10 | STI-S | 0.3375 | 0.0625 | 0.6739 | 0.0434 | 0 | 0.1565 | 0.048 | 0 | 0.2414 | 0.069 |
| 50 | KOTI | 50 | KOTI | 0.7694 | 0.6538 | 0.8269 | 0.439 | 0.32 | 0.5714 | 0.4166 | 0 | 0.7101 | 0.5264 |
| 50 | STI-K | 50 | STI-K | 0.6909 | 0.3056 | 0.8542 | 0.4837 | 0.1143 | 0.6441 | 0.47 | 0.0619 | 0.8321 | 0.6969 |
| 50 | STI-S | 50 | STI-S | 0.534 | 0.0938 | 0.6813 | 0.1003 | 0 | 0.1918 | 0.1139 | 0 | 0.3396 | 0.1229 |
| 100 | KOTI | 100 | KOTI | 0.8149 | 0.7238 | 0.8864 | 0.5985 | 0.4952 | 0.6804 | 0.664 | 0.5714 | 0.7097 | 0.6361 |
| 100 | STI-K | 100 | STI-K | 0.8396 | 0.8 | 0.8696 | 0.6203 | 0.5135 | 0.6897 | 0.6321 | 0 | 0.8217 | 0.7462 |
| 100 | STI-S | 100 | STI-S | 0.6407 | 0.4789 | 0.7381 | 0.2754 | 0 | 0.597 | 0.4131 | 0 | 0.6838 | 0.3269 |

**Smoking Task with Balanced Training Examples**

| N | Template | GatorTron | 95%LCI | 95%UCI | ClinicalBERT | 95%LCI | 95%UCI | BioGPT | 95%LCI | 95%UCI | ClinicalT5 | 95%LCI | 95%UCI |
| --- | --- | --- | --- | --- | --- | --- | --- | --- | --- | --- | --- | --- | --- |
| 0 | KOTI | KOTI | 0.1815 | 0.1815 | 0.1815 | 0.1643 | 0.1643 | 0.1643 | 0.1445 | 0.1445 | 0.1445 | 0.0667 | 0.0667 |
| 0 | STI-K | STI-K | 0.1517 | 0.1517 | 0.1517 | 0.0803 | 0.0803 | 0.0803 | 0.1428 | 0.1428 | 0.1428 | 0.0667 | 0.0667 |
| 0 | STI-S | STI-S | 0.128 | 0.128 | 0.128 | 0.0678 | 0.0678 | 0.0678 | 0.0872 | 0.0872 | 0.0872 | 0.0667 | 0.0667 |
| 1 | KOTI | KOTI | 0.3577 | 0.2423 | 0.4868 | 0.2511 | 0.1788 | 0.3024 | 0.2051 | 0.0854 | 0.3182 | 0.2694 | 0.2042 |
| 1 | STI-K | STI-K | 0.344 | 0.1849 | 0.4258 | 0.194 | 0.0492 | 0.3178 | 0.2369 | 0.1499 | 0.3264 | 0.2619 | 0.1545 |
| 1 | STI-S | STI-S | 0.3206 | 0.2243 | 0.3885 | 0.1889 | 0.0476 | 0.2812 | 0.2098 | 0.1396 | 0.2838 | 0.2626 | 0.1623 |
| 4 | KOTI | KOTI | 0.4321 | 0.3658 | 0.5322 | 0.281 | 0.2084 | 0.3489 | 0.2404 | 0.0862 | 0.3452 | 0.254 | 0.179 |
| 4 | STI-K | STI-K | 0.4012 | 0.2755 | 0.536 | 0.2976 | 0.2169 | 0.3558 | 0.268 | 0.2033 | 0.3615 | 0.3298 | 0.2341 |
| 4 | STI-S | STI-S | 0.3674 | 0.1245 | 0.7451 | 0.2755 | 0.192 | 0.3777 | 0.2347 | 0.178 | 0.3233 | 0.2441 | 0.2009 |
| 10 | KOTI | KOTI | 0.3776 | 0.1438 | 0.602 | 0.2637 | 0.14 | 0.3347 | 0.2144 | 0.0864 | 0.3033 | 0.2922 | 0.1824 |
| 10 | STI-K | STI-K | 0.4211 | 0.2911 | 0.6553 | 0.2763 | 0.1767 | 0.4413 | 0.2173 | 0.1601 | 0.2778 | 0.3217 | 0.2395 |
| 10 | STI-S | STI-S | 0.3403 | 0.2147 | 0.5919 | 0.251 | 0.1773 | 0.3524 | 0.278 | 0.1819 | 0.3696 | 0.311 | 0.2179 |

**Smoking Task with Random Training Examples**

| N | Template | GatorTron | 95%LCI | 95%UCI | ClinicalBERT | 95%LCI | 95%UCI | BioGPT | 95%LCI | 95%UCI | ClinicalT5 | 95%LCI | 95%UCI |
| --- | --- | --- | --- | --- | --- | --- | --- | --- | --- | --- | --- | --- | --- |
| 1 | KOTI | 1 | KOTI | 0.3473 | 0.2256 | 0.5899 | 0.2792 | 0.1833 | 0.3876 | 0.2159 | 0.0745 | 0.3118 | 0.2569 |
| 1 | STI-K | 1 | STI-K | 0.3112 | 0.1677 | 0.4 | 0.2575 | 0.0738 | 0.3543 | 0.2564 | 0.1395 | 0.3443 | 0.2886 |
| 1 | STI-S | 1 | STI-S | 0.2334 | 0.1299 | 0.3048 | 0.2338 | 0.0551 | 0.3395 | 0.2022 | 0.1586 | 0.2591 | 0.2711 |
| 4 | KOTI | 4 | KOTI | 0.3354 | 0.2645 | 0.4347 | 0.2961 | 0.2525 | 0.3602 | 0.2695 | 0.1374 | 0.3896 | 0.2858 |
| 4 | STI-K | 4 | STI-K | 0.3336 | 0.2524 | 0.4444 | 0.3105 | 0.2409 | 0.3932 | 0.2911 | 0.197 | 0.4304 | 0.3117 |
| 4 | STI-S | 4 | STI-S | 0.2963 | 0.2242 | 0.3678 | 0.2677 | 0.206 | 0.334 | 0.2692 | 0.1998 | 0.3058 | 0.2974 |
| 10 | KOTI | 10 | KOTI | 0.471 | 0.2179 | 0.7405 | 0.3167 | 0.2204 | 0.3742 | 0.3181 | 0.2 | 0.4155 | 0.311 |
| 10 | STI-K | 10 | STI-K | 0.4243 | 0.2876 | 0.6447 | 0.3326 | 0.2153 | 0.4122 | 0.3466 | 0.1993 | 0.4666 | 0.3471 |
| 10 | STI-S | 10 | STI-S | 0.3253 | 0.2172 | 0.6322 | 0.2984 | 0.191 | 0.3821 | 0.2537 | 0.1803 | 0.416 | 0.3426 |
| 50 | KOTI | 50 | KOTI | 0.3976 | 0.2769 | 0.5495 | 0.3464 | 0.2567 | 0.4718 | 0.2555 | 0.1921 | 0.3408 | 0.3226 |
| 50 | STI-K | 50 | STI-K | 0.3555 | 0.1985 | 0.5885 | 0.3354 | 0.2374 | 0.4066 | 0.3078 | 0.2479 | 0.4418 | 0.3137 |
| 50 | STI-S | 50 | STI-S | 0.3435 | 0.1643 | 0.6297 | 0.2465 | 0.1689 | 0.3405 | 0.2957 | 0.2251 | 0.3774 | 0.3051 |
| 100 | KOTI | 100 | KOTI | 0.275 | 0 | 0.5 | 0.23 | 0 | 0.5 | 0.1975 | 0 | 0.25 | 0.235 |
| 100 | STI-K | 100 | STI-K | 0.3067 | 0.2 | 0.5 | 0.205 | 0 | 0.25 | 0.21 | 0 | 0.25 | 0.24 |
| 100 | STI-S | 100 | STI-S | 0.2367 | 0 | 0.5 | 0.2008 | 0 | 0.3333 | 0.24 | 0 | 0.5 | 0.2017 |

**Appendix D: Token Number Distribution**

Table D 1 shows the average number of tokens per clinical notes in N2C2 obsity challenge, N2C2 smoking challenge and our dysmnorrhea dataset. It includes an estimate of the number of inference runs per clinical note required to process the whole input as described by Sivarajkumar and Wang (2022) and used in the HealthPrompt framework.

| Data-set | Average number of tokens per clinical note | SD | Proportion of clinical notes with >512 tokens | Estimated average number of inference runs per clinical note |
| --- | --- | --- | --- | --- |
| N2C2 obesity challenge | 1568 | 658 | 0.989 | 3.1 |
| N2C2 smoking challenge | 739 | 505 | 0.602 | 1.4 |
| Dysmenorrhea | 1011 | 278 | 0.983 | 2.0 |

D 1: Token Number Distribution. We calculate the average number of tokens per clinical note across the datasets and report the proportion of clinical notes with higher number of tokens with respect to the model’s token number limit.

**Appendix E: Number of Keyword Matches Across Datasets**

E1: Distribution of the number of keyword matches across datasets
